# Ethosome-based drug delivery system for precision targeting and tumour microenvironment reprogramming in non-invasive skin cancer therapy

**DOI:** 10.1186/s11671-026-04738-9

**Published:** 2026-10-22

**Authors:** Madhavi Porwal, Sathvik Belagodu Sridhar, Javedh Shareef, Tarun Wadhwa, Balamurugan Balusamy, Phool Chandra, Rishabha Malviya

**Affiliations:** 1 Department of Pharmacy, School of Medical and Allied Sciences, Galgotias University, Greater Noida, Uttar Pradesh 201308 India; 2 RAK College of Pharmacy, RAK Medical & Health Sciences University, Ras Al Khaimah, United Arab Emirates; 3 College of Pharmacy, Dubai Medical University, Dubai, United Arab Emirates; 4 School of Engineering and IT, Manipal Academy of Higher Education, Dubai Campus, Dubai, United Arab Emirates; 5 Teerthanker Mahaveer College of Pharmacy, Teerthanker Mahaveer University, Moradabad, Uttar Pradesh 244001 India

**Keywords:** Ethosomes, Non-invasive skin cancer, Tumour microenvironment, Precision targeting, Transdermal drug delivery, Nanomedicine

## Abstract

The non-invasive skin cancer, especially that of the basal cell carcinoma and squamous cell carcinoma, is an increasing global health burden. Traditional methods of treatment, such as surgery, cryotherapy, and topical chemotherapeutics, are all limited by poor penetration into the skin, non-specific drug delivery, cosmetic destruction, and tumour reappearance. Moreover, the tumour microenvironment (TME) significantly contributes to disease progression through inflammation, angiogenesis, immunosuppression, and therapeutic resistance, underscoring the need for more advanced, targeted treatment methods. The purpose of this review is to critically analyse the applications of the ethosome-based drug delivery system in the precision targeting and reprogramming of the TME in the treatment of non-invasive skin cancer. Ethosomes have better skin permeability and local drug delivery than standard topical systems. They also facilitate easy access to a wide variety of therapeutic agents, allowing specific interactions with tumour tissue and other critical components of the TME, leading to better therapeutic effects with fewer off-target effects. The ethosome-based drug delivery system is an attractive non-invasive therapeutic platform for managing skin cancer by efficiently reprogramming the TME via transdermal delivery. Although they have preclinical evidence supporting their potential, they need additional clinical validation and regulatory standardisation to be successfully translated into clinical use.

**Graphical abstract:**

**Figure:**
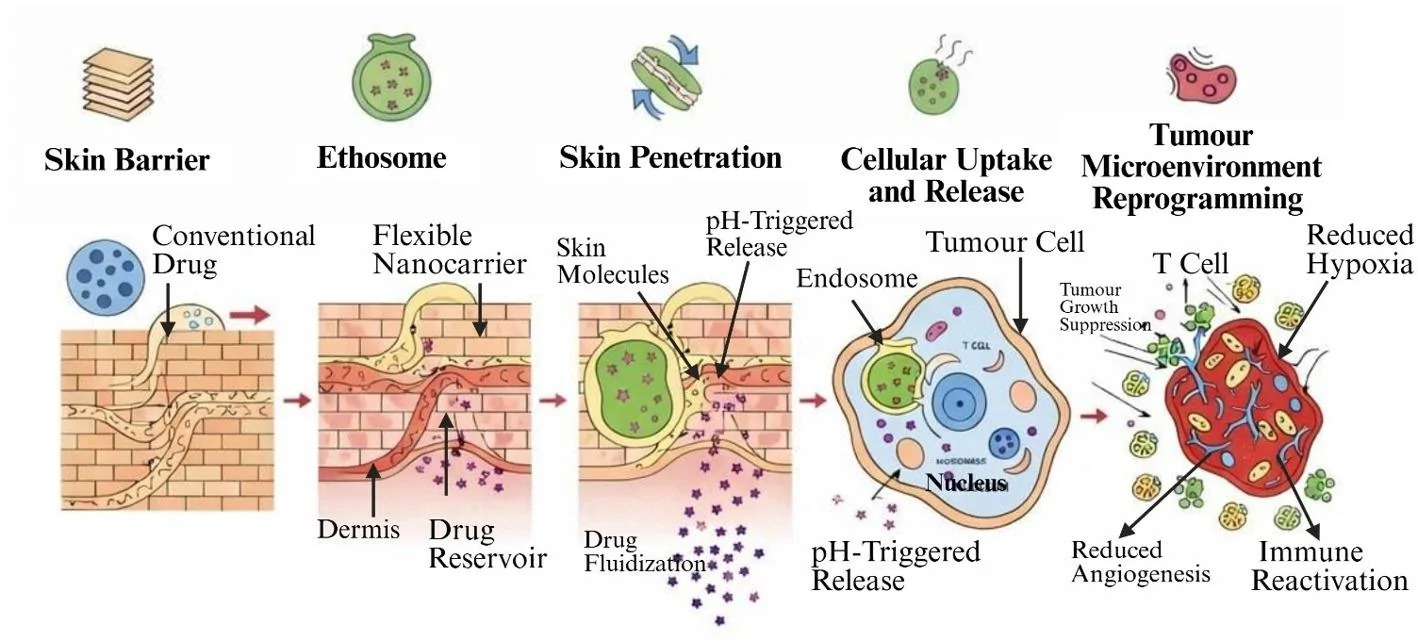

## Introduction

Transdermal drug delivery has become an appealing alternative to traditional routes of administration because of its ability to provide sustained drug release, enhance patient compliance, and bypass hepatic first-pass metabolism. Despite these benefits, the clinical usefulness of transdermal systems remains greatly limited by the skin’s inherent resistance, which restricts the absorption of a wide variety of therapeutic molecules. Over the last few years, significant research has been conducted on the management of advanced carrier systems to overcome these constraints [1, 2]. Lipid-based vesicular systems have shown great potential, as they are biocompatible and can enhance drug permeation. Nevertheless, traditional vesicular delivery vehicles like liposomes tend to have limited penetration, which limits their practical use in transdermal delivery. To overcome these challenges, ethosomes, a derivative vesicular system with a large ethanol content, have been proposed. Besides providing flexibility to the vesicles, ethanol also increases vesicle interactions with skin lipids, promoting better drug penetration and greater tissue penetration. This characteristic has made ethosomes a better choice than conventional vesicular carriers in the delivery of drugs through the skin. This review comprehensively discusses the potential of ethosomal drug delivery systems for targeting the TME in non-invasive skin cancers, with particular emphasis on precision targeting, nanocarrier comparison, and future integration with artificial intelligence for optimised therapeutic design [3].

### Limitations of conventional topical and systemic therapies

The existing modalities utilised for non-invasive skin malignancies include surgery, cryotherapy, photodynamic therapy, topical chemotherapeutics, and, in advanced cases, systemic medicines. The gold standard is still maintained for the localised lesions that are well curable. However, it is mostly accompanied by serious limitations such as scarring, morbidity of the procedure and less frequent use on bigger or more aesthetically sensitive locations. Although topical therapies are non-invasive and convenient, they have severe flaws. The stratum corneum is the first biophysical barrier, the outermost layer of the skin, and the most effective at resisting medication penetration. This obstacle hinders various types of treatment, particularly those with poor molecular properties (e.g., high polarity or high molecular weight). This contributes to the observation that traditional topical agents frequently result in low drug delivery to the target site and, consequently, to non-optimal treatment rates [4, 5]. Although systemic medicines are capable of reaching higher drug levels, they have systemic adverse effects and off-target toxicity, which could restrict their use over time and adherence to the patient. This is of particular concern in instances such as skin malignancies. The therapeutic index of a drug is the ratio of its therapeutic effect to its toxicity, and it should be fine-tuned to ensure successful treatment without compromising healthy tissues. The necessity to obtain a high concentration of anticancer drugs at the local level and minimum exposure to the systemic circulation has caused the appearance of research on innovative drug delivery methods that could be employed to bypass the stratum corneum and increase the bioavailability of anticancer drugs at the site of lesion of the disease [6].

### Rationale for nanocarrier-based transdermal drug delivery

Systems based on nanocarriers have been demonstrated in dermatology and oncology to circumvent the limitations of traditional treatments. The systems improve the solubility of the drug, stabilise volatile compounds, and can offer controlled or controlled-release delivery of drugs over time, flattening the pharmaceutical site of action by utilising nanoscale size, variant surface characteristics, and encapsulating characteristics. The transfer of drugs into the skin through the relatively thin stratum corneum can be improved from a mechanistic perspective by interactions between the nanocarrier and the skin, which can change permeation pathways either temporarily by altering lipid structures or permanently by permitting diffusion of molecules between cells. Incorporating chemotherapeutic drugs into nanocarriers may significantly improve their therapeutic efficacy and reduce systemic exposure and subsequent side effects, since most of them are not readily soluble in water or are rapidly degraded [7].

One category of nanocarrier systems that has been explored and shown promise is the vesicular carriers (liposomes, transfersomes, ethosomes, etc.), as they have the structural capability to match biological membranes and can be loaded with a broad range of drug classes. Not all transfersomes can, however, be formed by vesicles; although flexible, these can still be hindered by stability concerns, and normal liposomes may at times show poor penetration. Conversely, ethosomes are highly versatile for transdermal or dermal delivery of a therapeutic payload, as they can accommodate high amounts of ethanol within the lipid vesicles. Traditional vesicular systems such as liposomes, niosomes, and transfersomes have drawbacks regarding penetration depth, stability, and targeted delivery. These shortcomings have led to the development of more sophisticated systems, such as ethosomes, that are more transdermal and therapeutic [8].

### Emergence of ethosomes in dermal oncology

A lipid vesicle nanocarrier is called an ethosome, and it is mostly composed of phospholipids, water, and high levels of ethanol (usually 20–45%). The ethosomes are peculiar due to their high ethanol concentration, which increases vesicle fluidity and deformability and interferes with the organisation of stratum corneum lipids, allowing them to travel deeper into the epidermis [9].

The ethosomal vesicles deform and cross small intercellular spaces to increase transdermal flux and the successful delivery of encapsulated medications into the epidermis and dermis. Non-invasive tumours of the skin, compared with traditional liposomes, are in most cases more rigid and fixed to the superficial layers. This increased permeability can be linked to ethanol’s ability to fluidise lipid bilayers and enhance vesicle flexibility, thereby enabling vesicles to avoid the resistance that traditional carriers face. This aspect, coupled with the ability to simultaneously store hydrophilic and lipophilic drugs and their higher permeability, enables the use of ethosomes as chemotherapeutic agents, immunomodulatory agents, and gene therapy vectors. The use of ethosomes is especially beneficial because of their non-invasive delivery and their ability to achieve high local drug concentrations with minimal systemic exposure, which is particularly appealing in the cutaneous oncology market .

## Tumour microenvironment (TME) in skin cancer

### Cellular components of skin cancer TME

**Fig. 1:**
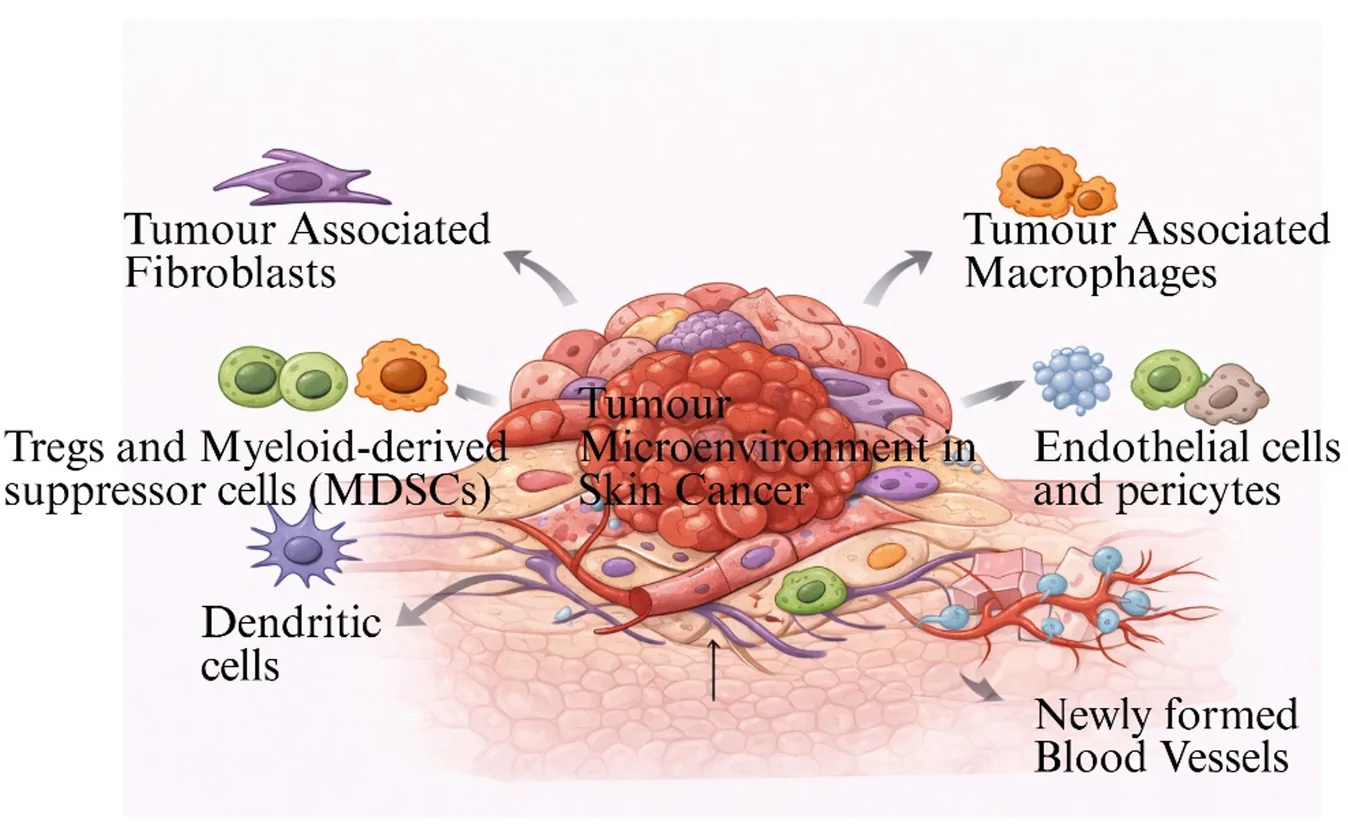
Tumour microenvironment in skin cancer

TME represents a complex ecosystem comprising malignant cells and a vast array of stromal and immune cells that interact to control tumour invasion, growth, and treatment in skin malignancies. Cancer-associated fibroblasts (CAFs), endothelial cells, pericytes, and immunological infiltrates, including tumour-associated macrophages (TAMS), myeloid-derived suppressor cells (MDSCs), regulatory T cells (Tregs), and dendritic cells (DCs), are important cell types. These cells actively engage and communicate with cancer cells via cytokines, chemokines, and growth factors, thereby creating a pro-tumourigenic environment rather than becoming mere spectators [10]. For example, TAMs tend to be immunosuppressive in the M2-like phenotype (which inhibits cytotoxic immunity and promotes cancer development), and CAFs release ECM components, proteases, and signalling molecules (which stimulate tumour cell proliferation, invasion, and metastasis). Angiogenesis is mediated by endothelial cells and pericytes, which are necessary to supply the tumour with nutrients and facilitate metastasis. Immune cells can mediate anti-tumour immunity or be immunosuppressive and unresponsive to treatment, depending on their subtype and activation state. It is a combination of these cells that forms a microenvironment that enables tumours to thrive and survive [11] (Fig. 1).

### Role of hypoxia, inflammation, and ECM remodelling

The TME in solid tumours, particularly skin malignancies, is also characterised by hypoxia or low oxygen tension, which is caused by the expansion of the fast-growing tumour cell bulk that utilises factors of production, in this case, blood. Hypoxia has been known to stabilise hypoxia-inducible factors (HIFs), which mediate transcriptional programmes that favour angiogenesis, metabolic reprogramming, survival innervation, and immunological regulation. Hypoxic conditions shift the metabolic profile of tumours toward glycolysis, the so-called Warburg effect. They result in the accumulation of lactate, acidification of the microenvironment, and the blockade of anti-tumour immunity, thereby supporting the survival and proliferation of cancer cells [12].

The tumour and stromal cell-secreted pro-inflammatory cytokines (e.g., IL-6, TNF-α) also favour immunological and stromal signals, thereby enhancing tumour progression. Chronic inflammatory signalling encompasses inflammatory remodelling of the vascular system and immunopathogenesis [13]. Another interesting aspect of TME is that it remodels ECM proteins by activating proteolytic enzymes, such as matrix metalloproteinases (MMPs), which in turn alter the structural composition of the ECM, allowing invasion and metastasis of cancer cells. Hypoxia stimulates ECM remodelling by HIF action and collagen-modifying enzyme and crosslinking factor activation to achieve a stiff ECM and fibrotic and invasion-permissive environment. The interaction with the modified extracellular matrix also alters cell signalling by interacting with modified integrins and other receptors, which control cell migration, proliferation and survival [14].

### Immune suppression and angiogenesis in skin tumours

There is a wide variety of strategies that immunosuppressive TME and immune evasion use. It is stromal signalling and hypoxia that promote the expression of immunological checkpoint molecules, e.g., PD-L1 and CTLA-4, which bind T-cell receptors, thereby triggering T-cell fatigue and repressing anti-tumour immune responses. To further inhibit cytotoxic T-cells and antigen presentation, the TME also attracts and expands immunosuppressive cell types, such as Tregs, MDSCs, and M2-polarised TAMs, which secrete IL-10, TGF-β, and arginase [15]. The other TME characteristic of skin tumours is angiogenesis or the growth of new blood vessels, which is stimulated by hypoxia-stimulating molecules such as vascular endothelial growth factor (VEGF). The greater the angiogenesis, the greater the chances of metastasis and the supply of tumours with oxygen and other factors that support their development. Nevertheless, the tumour vessels tend to be irregular and porous, which elevates resistance to therapy, immune cell exclusion, and hypoxia [16].

### TME as a therapeutic target

TME is now an important cancer treatment target, especially in skin cancer, as it is a major contributor to immunity evasion, resistance to treatment and tumour expansion. To target the TME to regain effective anti-tumour immunity, the supportive stromal networks must be destabilised, the aberrant vasculature remodelled, hypoxia alleviated, and the immunosuppressive networks re-educated. Anti-angiogenic drugs, in which case, serve to heal normal blood arteries and decrease hypoxia, and immune checkpoint inhibitors, in which case, are efforts to revitalise exhausted T cells. They can control hypoxia and mitigate immune suppression (and predispose tumours to immunotherapy and other interventions) through the use of HIF inhibitors and oxygen-generating nanocarriers, among others [17, 18].

Another interesting opportunity for TME targeting lies in nanocarrier-based drug delivery systems that have the potential to increase drug concentration within the tumour, enhance penetration, and enable targeted delivery of drug therapy agents such as immunomodulators, chemotherapeutics, or hypoxia-responsive drugs. These plans are intended to transform the TME into an immunosuppressive niche for tumour eradication and improved treatment outcomes [19] (Table 1).

**Table 1:** Key components of tumour microenvironment

TME component	Major elements	Functional role in skin cancer progression	Therapeutic relevance	Reference
Cellular components	Cancer-associated fibroblasts (CAFs), tumour-associated macrophages (TAMs), Tregs, MDSCs, endothelial cells and pericytes	Cytokine and growth factor secretion promote tumour growth, invasion, immune evasion and angiogenesis	Tumour-stroma interactions can be interfered with by targeting CAFs and immune cells	[20, 21]
Hypoxia	Low oxygen tension, HIF-1α, HIF-2α	Causes angiogenesis, reprogramming of metabolic activity, suppression of immunity and resistance to treatment	Enhanced treatment sensitivity is achieved through hypoxia-targeted therapies	[22, 23]
Inflammation	Pro-inflammatory cytokines (IL-6, TNF-α), chemokines	Maintains tumour growth, attracts immune suppressive cells, and facilitates the development of metastasis	Anti-inflammatory measures can help in slowing down tumour progression	[24]
Extracellular matrix (ECM)	Collagen, fibronectin, laminin, proteoglycans	ECM remodelling increases tumour invasion, stiffness-mediated signalling and metastasis	Drug penetration is enhanced in ECM-targeted delivery	[25, 26]
Immune suppression	Tregs, MDSCs, PD-L1, IL-10, TGF-β	Inhibits Th1 lymphocytes, making the immune system weak	Antitumour immunity is restored with the use of immune checkpoint inhibitors	[27, 28]
Angiogenesis	VEGF, FGF, abnormal vasculature	Promotes tumour development, the supply of nutrients and metastasis	Tumour vessels are normalised by anti-angiogenic therapy	[29]
TME as therapeutic target	Immunology, vascularity and hypoxia systems	Combination therapy increases treatment efficacy and decreases resistance	Localised TME modulation is made possible using nanocarriers	[30, 31]

## Challenges in transdermal drug delivery for skin cancer

### Skin barrier properties

The stratum corneum is the primary biological barrier to effective transdermal drug delivery in the treatment of skin cancer. Structurally, it is composed of flattened, enucleated corneocytes fixed in a highly organised lipid matrix mainly composed of ceramides, cholesterol, and long-chain fatty acids. This is a lipid-filled extracellular space that forms closely spaced lamellar bilayers that severely restrict molecular diffusion. This reticence represents a significant impediment to the delivery of anticancer agents, which can be hydrophilic, macromolecular, or chemically labile, from a therapeutic perspective [32]. In addition, epidermal architecture can be disturbed in pathological skin disorders like non-melanoma skin cancers that, however, do not often affect the stratum corneum to such a degree that drugs can readily penetrate it. Consequently, the majority of topically applied anticancer drugs fail to reach therapeutic concentrations in tumour tissue. Bypassing the stratum corneum barrier without inducing irreversible skin alterations is a key challenge and the primary justification for technological advances in lipid-based nanoparticles, such as ethosomes, which can solubilise skin lipids and enhance drug diffusion [33, 34].

### Poor drug penetration and retention

Alongside low permeation through the stratum corneum, traditional transdermal devices are characterised by low penetration of drugs into the deep layers of the skin and by their retention in tumour tissue. A good number of non-invasive skin cancers, such as basal cell carcinoma and squamous cell carcinoma, spread beyond the superficial epidermis into deeper layers, becoming the focus of long-term exposure to drugs at certain levels. Nevertheless, traditional creams, gels, and ointments can be quickly diffused away from the point of application, enzymatically degraded, or absorbed by the body. This results in short-lived drug levels, insufficient to kill tumour cells or modify the tumour microenvironment. Weak retention of drugs also creates the need to administer the drugs frequently, which decreases patient compliance and increases the risk of adverse reactions. For efficient skin cancer treatment, the drug delivery system should have a long residence time and controlled release to the tumour, which traditional formulations cannot offer simultaneously, but this may be possible with the use of nanovesicle carriers such as ethosomes [35, 36].

### Non-specific drug distribution and toxicity

One of the main drawbacks of current transdermal and topical treatments of skin cancer is the absence of spatial and cellular specificity. After a drug crosses the skin barrier, it tends to spread non-selectively into adjacent healthy tissue or enter the systemic circulation via dermal capillaries. It is a non-specific distribution that may lead to local skin irritation, inflammation, ulceration, and damage of healthy keratinocytes and fibroblasts. Cytotoxic agents may lead to off-target toxicity with systemic exposure, undermining localised therapy. In non-invasive skin cancers, the lesions are limited and physically accessible; non-specific extravasation of drugs is especially undesirable, as it affects cosmetic results and patient well-being. Thus, the localisation of drug concentration within tumour tissue and the minimal exposure of normal skin are among the most important unmet requirements in dermal oncology. It is necessary to address this issue using precision-engineered nanocarriers that can be selectively deposited into tumours and released under control [37].

### Need for precision targeting

**Fig. 2:**
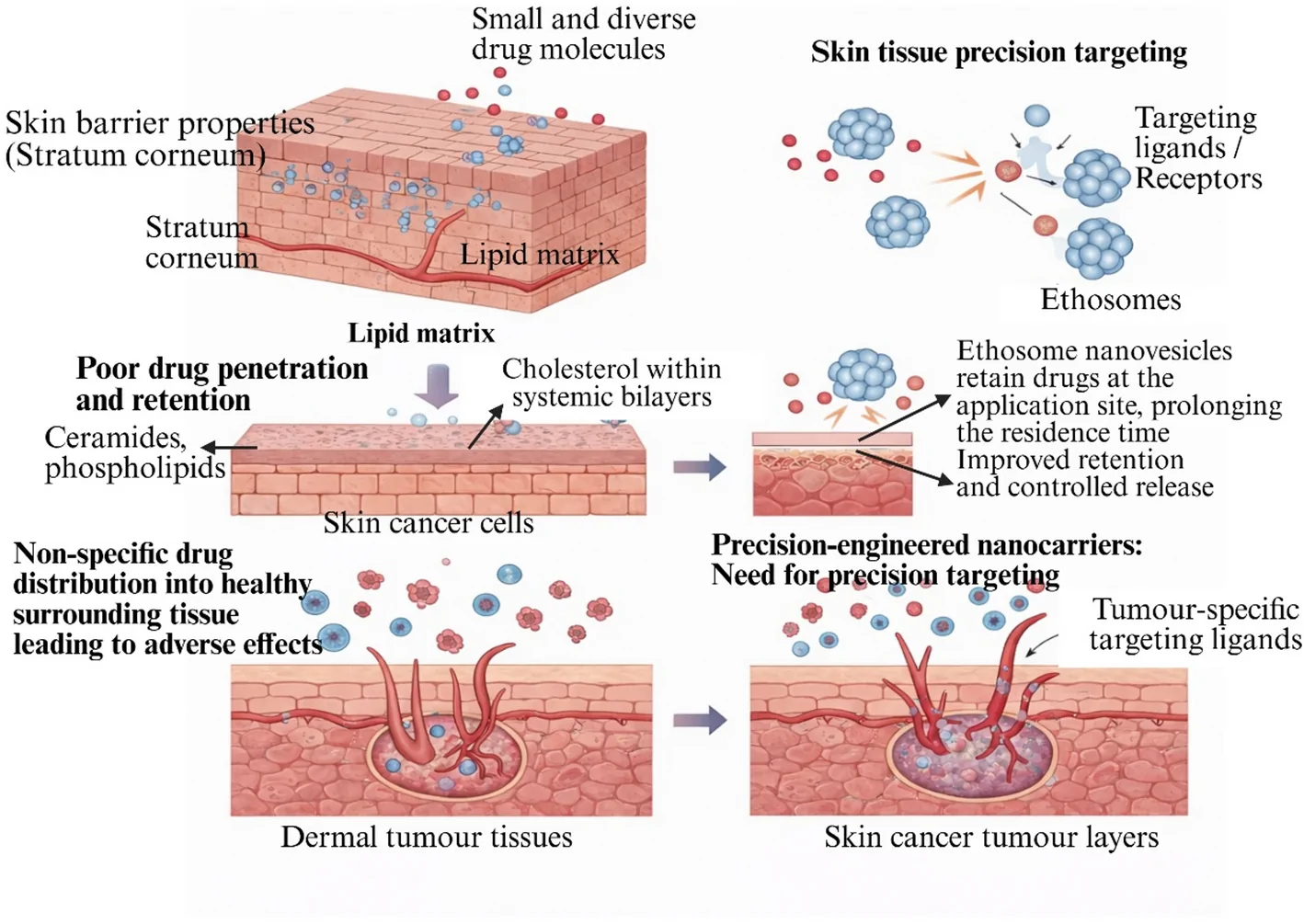
Challenges in transdermal drug delivery for skin cancer therapy

The high complexity of skin cancer biology and the TME underscores the urgent need for precision-targeted transdermal drug delivery systems. Precision targeting not only entails selective delivery of drugs to tumour cells but also modulates the microenvironment surrounding the tumour, including inflammatory mediators, immune cells, hypoxic cells, and abnormal vasculature. These microenvironmental cues are inaccessible to conventional topical therapies, thus leading to poor therapeutic responses. Lipid-based vesicular systems, which are advanced nanocarriers, have the potential to be utilised in targeted delivery due to increased permeability and selective retention, and can be modified with receptors to enable receptor-mediated uptake. With their distinctive high ethanol content and flexible lipid bilayer, ethosomes are deemed particularly suitable for precision skin cancer therapy. They can penetrate deeply into the skin, concentrate at the tumour site, and potentially be used for TME reprogramming. Therefore, the concept of precision targeting represents a paradigm shift from passive drug diffusion to site-specific, localised therapy for the treatment of non-invasive skin cancer [31, 38] (Fig. 2).

## Ethosomes: a novel vesicular drug delivery system

### Definition and composition of ethosomes

**Fig. 3:**
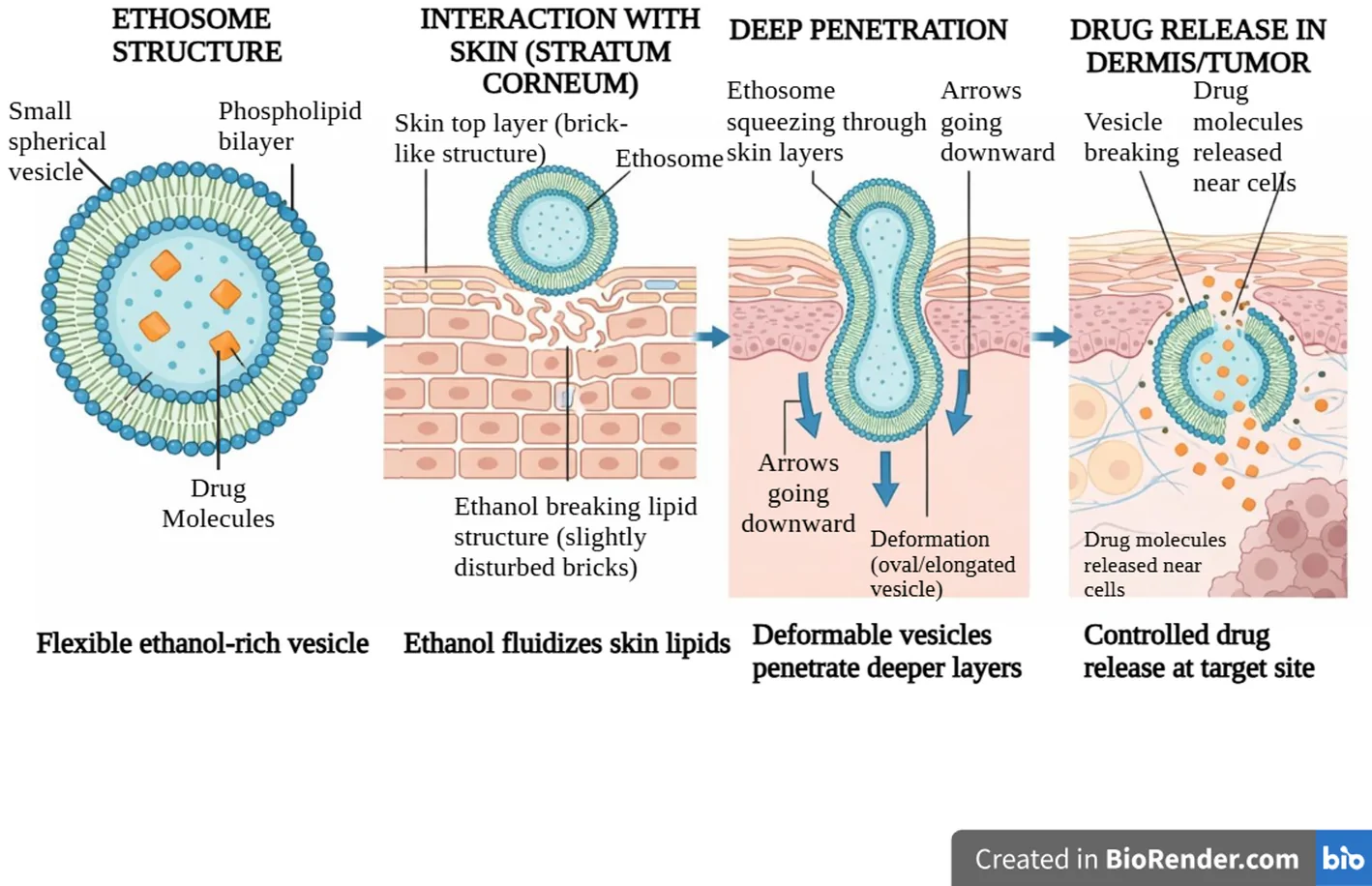
Mechanism of skin drug delivery via ethosomes

Ethosomes are novel lipid-based vesicular drug delivery systems specifically designed to enhance drug transport across transdermal and dermal routes. They are differentiated from traditional vesicles by their high ethanol concentration, usually 20–45 per cent, which is included in the phospholipid bilayer. Ethanol, glycoses, water, cholesterol, and penetration enhancers are optional constituents of the basic composition of ethosomes, which also contains phospholipids (e.g. phosphatidylcholine) and ethanol. Ethanol softens the vesicle membrane, making the vesicles highly deformable and able to penetrate deeper layers of the skin. Also, ethanol enhances the solubility of both hydrophilic and lipophilic drugs, thereby increasing drug-loading efficiency and improving stability. Although the ethosomes have shown high drug loading and penetration rates, their functionality is very formulation-dependent. Fluctuations in ethanol concentration, lipid composition, and vesicle size can significantly affect stability and drug leakage. Furthermore, the degree of controlled release and reproducibility across various formulations remains inadequately standardised, although permeability has improved. Thus, despite the evident benefits of ethosomes compared to traditional carriers, their optimisation should be undertaken with a prudent balance between permeability enhancement and vesicle stability [39, 40] (Fig. 3).

### Role of ethanol in membrane fluidisation

The most crucial functional ingredient responsible for the outstanding transdermal activity of ethosomes is ethanol. At the vesicular level, ethanol reacts with the phospholipid head groups, decreasing lipid packing density and elevating bilayer fluidity. This leads to very flexible and deformable vesicles that interact with stratum corneum lipid and pass through the thin intercellular spaces in the stratum corneum. Ethanol interferes with the highly ordered lipid lamellae of the stratum corneum at the skin level by extracting and fluidising epidermal lipids, thereby reducing barrier resistance. This two-pronged process of vesicle softening and disruption of skin lipids creates a synergistic effect on drug permeation. This ethanol-based fluidisation of the membrane can be used in the treatment of skin cancer to enhance deeper penetration of the drug into skin layers containing tumours and to increase its accumulation in the tumour. In addition, ethanol can enhance the uptake of intracellular drugs by tumour cells, leading to increased cytotoxic effects and possible remodelling of the tumour microenvironment. Nevertheless, a high ethanol content can damage the vesicles and cause skin irritation, underscoring the significant need to optimise the ethanol content in the formulation to ensure safety in clinical translation [6].

### Types of ethosomes

Ethosomes have been developed in various forms to further enhance penetration into the skin, stability, and therapeutic effect. Classical ethosomes are made of phospholipids, ethanol, and water and are commonly utilised for basic transdermal applications. Binary ethosomes contain an additional penetration enhancer, e.g., propylene glycol or isopropyl alcohol, which further increases permeation and drug retention in the skin. Transethosomes are a higher-order system that incorporates the benefits of ethosomes and transfersomes, incorporating edge activators such as surfactants. This alteration generates ultra-deformable vesicles with enhanced penetration and elasticity. Transethosomes are effective in delivering anticancer drugs deeper into the skin and greater targeting of tumour tissue. They are especially promising in targeted therapy of the tumour and TME modulation in non-invasive skin cancers due to their capacity to circumvent biological barriers and adjust to the TME [41, 42].

### Comparison with liposomes, niosomes, and transfersomes

**Fig. 4:**
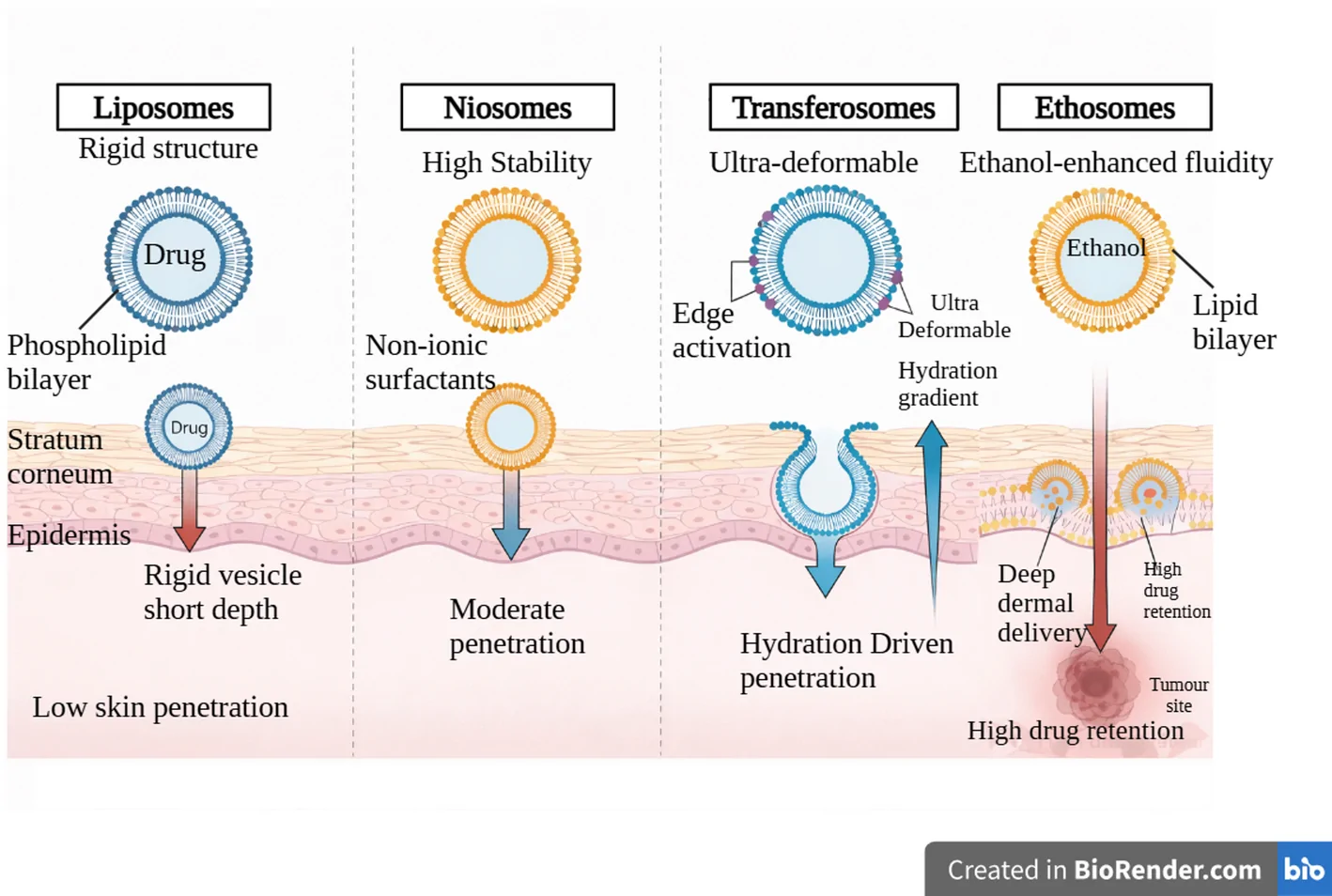
Comparative analysis of vesicular drug delivery systems for transdermal cancer therapy

Ethosomes exhibit significant advantages over conventional vesicular drug delivery systems, such as liposomes, niosomes, and transfersomes, particularly in skin permeation and therapeutic efficacy. While liposomes are composed primarily of phospholipid bilayers, they lack sufficient flexibility and penetration capacity, thereby limiting their effectiveness in transdermal delivery [43]. Niosomes, formed using non-ionic surfactants, offer improved stability and cost-effectiveness but still face challenges in penetrating the intact stratum corneum. Transfersomes, which include edge activators, demonstrate enhanced deformability and improved penetration compared to liposomes and niosomes; however, their performance often depends on external factors such as hydration gradients. In contrast, ethosomes contain a high concentration of ethanol, which plays a crucial role in disrupting the lipid organisation of the stratum corneum and increasing vesicle fluidity. This dual mechanism allows ethosomes to penetrate deeper into skin layers and deliver drugs more efficiently. As a result, ethosomes are considered highly promising carriers for targeted and non-invasive treatment of skin cancers [44]. In general, although each vesicular system has potential for transdermal drug delivery, its clinical applicability differs. The liposomes and niosomes are safe and stable, but have low penetration efficiency. Transfersomes enhance deformability, but have challenges in formulation and stability. In comparison, ethosomes offer better penetration due to ethanol-induced lipid fluidisation, but the system has limitations for clinical translation due to the risk of skin irritation, formulation instability, and regulatory disqualifiers. Thus, although ethosomes have potential benefits, they still need to be optimised further and validated on a large scale before they can be used in a routine clinical setting [45] (Table 2, Fig. 4).

**Table 2:** Comparative analysis of vesicular drug delivery systems

System	Composition	Mechanism of action	Advantages	Limitations	Clinical relevance	Reference
Liposomes	Phospholipid bilayer vesicles	Passive diffusion, limited penetration	Biocompatible, non-toxic	Poor skin permeation, low stability	Limited use in topical formulations	[43]
Niosomes	Non-ionic surfactant-based vesicles	Improved interaction with skin lipids	Stable, cost-effective	Moderate penetration ability	Used in dermal and cosmetic applications	[44, 45]
Transfersomes	Lipids with edge activators	Highly deformable vesicles enabling deeper penetration	Enhanced flexibility and permeability	Performance depends on hydration conditions	Advanced transdermal drug delivery	[46]
Ethosomes	Phospholipids with high ethanol content	Ethanol-induced lipid fluidisation + enhanced vesicle flexibility	Superior skin penetration, targeted delivery	Possible irritation, stability concerns	Promising for non-invasive skin cancer therapy	[47]

## Mechanism of skin penetration and cellular uptake of ethosomes

### Enhanced permeation and deep dermal delivery

The ethosome has been reported to exhibit increased permeation across the stratum corneum and delivery to the deeper layers of the epidermis and dermis following lipid interaction. It is easy to permeate the slender intercellular spaces that are inaccessible to the rigid carriers, such as the traditional liposomes, because they possess soft and deformable vesicles. This action is also enhanced by ethanol, which increases skin permeability and facilitates the migration of vesicles through the transepidermal pathway. Owing to this, ethosomes can deliver drugs to deeper regions of the skin, including the basal epidermis and upper dermis, and to non-melanoma skin cancers, while melanoma in situ often occurs. The depth and homogeneity of drug distribution vary across skin types and pathological conditions, even with improved dermal deposition. Moreover, the majority of evidence is based on in vitro or ex vivo experiments, which do not provide maximum predictability of in vivo performance. This demonstrates the need for additional clinically applicable models to confirm the true therapeutic benefit of ethosomal delivery [40–48].

### Endocytosis-mediated cellular internalisation

Once they reach accessible skin layers, ethosomes interact directly with both cancer and stromal cells within the tumour microenvironment. Internalisation of ethosomes via endocytosis occurs primarily through energy-dependent processes, including clathrin-mediated endocytosis, caveolae-mediated endocytosis, and macropinocytosis. The nanoscale size, lipidic constituents, and surface characteristics of ethosomes render them effective for recognition and uptake by tumour cells. Ethanol-induced membrane fluidisation also enhances the contacts between vesicles and cell membranes, through which food vesicles are internalised. Uptake will be efficiently achieved via endocytosis, enabling the intracellular accumulation of chemotherapeutic agents, antioxidants, and immunomodulators in the context of skin cancer. This has been made possible through the delivery of drugs to intracellular sites, thereby enhancing drug bioavailability at the cellular scale, circumventing drug resistance pathways, and enhancing therapeutic selectivity. Also, the uptake of immune cells by the TME can contribute to immune modulation and re-education of tumour-associated inflammatory responses [45].

### Intracellular drug release mechanisms

**Fig. 5:**
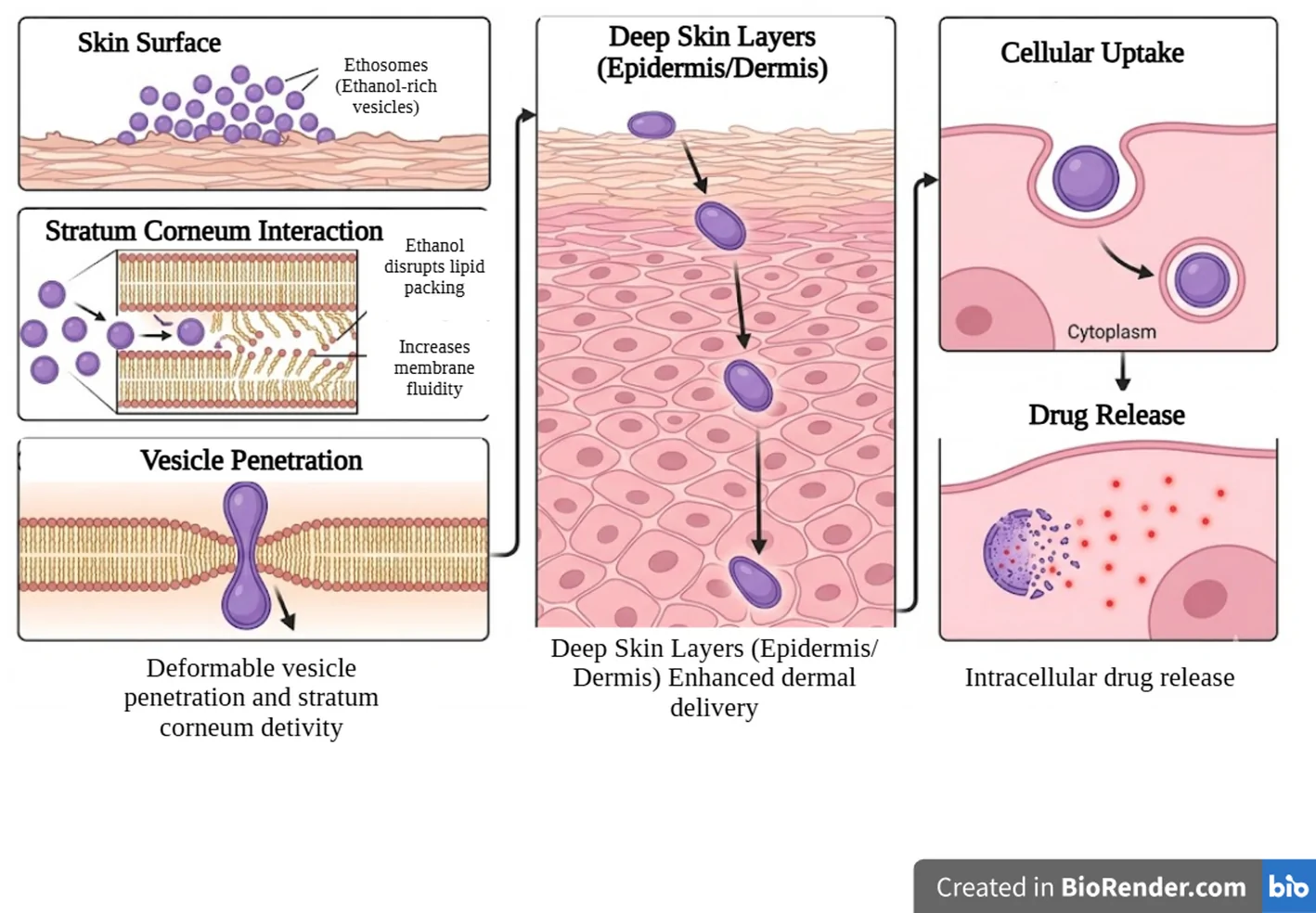
Mechanism of skin penetration and cellular uptake of ethosomes

The drug payload is released from the ethosomes following regulation of cellular events after internalisation, which is critical to the treatment effect. If the ethosomes are sequestered by endosomes or lysosomes, the acidic pH and enzymatic activity degrade the phospholipid bilayer of the ethosomes, causing the vesicles to rupture and release their drugs. Ethanol can also be used to destabilise membranes and has been shown to facilitate endosomal escape, thereby helping drugs reach their cytosolic and nuclear destinations. Released anticancer compounds can induce apoptosis, prevent cell growth, inhibit angiogenic signalling, and modify the inflammatory microenvironment of the tumour. This controlled cytosolic release has been applied in the non-invasive treatment of skin cancer to reduce off-target toxicity and ensure that the maximum effect is directed to the tumours. Also, reprogramming of the TME enables intracellular delivery of agents that modulate hypoxia, oxidative stress, or immune signalling, thereby improving therapeutic outcomes [38, 49] (Fig. 5).

## Ethosomes for precision targeting in skin cancer therapy

### Passive targeting via enhanced skin permeation

Ethosomes may achieve passive targeting due to their inherent ability to penetrate deep into the skin layers without the need for an active guidance mechanism. The high concentration of ethanol liquefies the lipid matrix of the stratum corneum, thereby loosening tight lipid packing and decreasing resistance to diffusion, resulting in passively accumulating ethosomes in the epidermis and dermis, sites of skin cancer development [50]. This passive accumulation is also enhanced by the fact that ethosomal bilayers are soft and compliant, allowing the vesicles to pass through narrow intercellular pathways and achieve greater local drug deposition than traditional vesicles such as liposomes or niosomes. Research has demonstrated that ethosomal carriers are associated with much higher permeation flux, deeper drug penetration, and dermal retention, thereby enhancing therapeutic efficacy and decreasing systemic absorption and toxicity. Such passive delivery will be important in the context of selective therapy of skin cancer, in which chemotherapeutic agents or immunomodulators targeted to pathologically relevant skin layers are concentrated, a critical factor for local cytotoxicity and tumour suppression [51].

### Ligand-functionalized ethosomes for active targeting

Although passive mechanisms enhance local drug accumulation, ligand-functionalized ethosomes offer greater selectivity through active targeting. Surface modification of ethosomes using specific ligands, i.e., peptides (e.g., RGD), folate, antibodies, or hyaluronic acid (HA), facilitates the detection of tumour-associated receptors overexpressed on cancer cells. Indicatively, vesicles covered with hyaluronic acid increased the cellular intake in tumour models by a large measure relative to unmodified vesicles, which resulted in greater cytotoxic activity and greater concentrations of drugs in tumour spheroids. These results demonstrate that ligand-mediated interactions can selectively target ethosomal carriers to tumour cells and enhance the delivery of therapeutic payloads. Despite the emergence of direct applications in skin cancer models, similar ligand-targeting approaches already established in breast or melanoma models form the basis of ethosome bioengineering strategies specific to overexpressed receptors in non-invasive skin cancers [52].

### Targeting cancer cells versus normal skin cells

The ability of ethosomes to distinguish malignant from normal skin cell uptake mechanisms is critical for the precision targeting of ethosomes. Altered membrane fluidity, overexpressed surface receptors, and a dysregulated microenvironment are common in tumour cells, unlike in normal keratinocytes. Owing to their flexible ethanol-rich bilayer, ethosomes behave differently with these cell types: cancer cells are more readily adherent to and taken up by vesicles because of their altered membrane properties and metabolism. Also, pH-sensitive or receptor-responsive ligands can be added to selectively deliver the drug to the slightly acidic tumour microenvironment, further increasing selectivity. This implies that the therapeutic agents reach their targets more effectively in cancer cells without exposing the surrounding healthy skin, thereby minimising collateral cytotoxicity and skin functions. Both in vitro and ex vivo investigations using ethosomal carriers to incorporate anticancer agents have shown varying cytotoxicity, with a preference for tumour cell death and minimal effects on normal skin cells, highlighting their potential as precise therapeutic agents [53].

### Role in reducing off-target effects

The therapeutic benefit of using ethosomes in skin cancer is closely linked to their ability to reduce off-target effects. Ethosomes can also deliver anticancer agents directly to the tumour-bearing layers of the skin, thereby focusing drug delivery in the area where the drug is most required and reducing diffusion to healthy tissue or body circulation. The permeable vesicular architecture and ethanol-induced lipid fluidisation enable high rates of deep dermal penetration, long-term retention, and controlled release of the payload, thereby decreasing the rate and extent of drug exposure to non-target cells. This innate targeting effect can increase the therapeutic index of cytotoxic agents and prevent non-target injury to normal skin cells, thereby reducing the risk of irritation, inflammation, and systemic toxicity. Also, vesicular encapsulation stabilises labile drugs, prevents their premature degradation, and enables the gradual release of drugs into the intracellular pathways of tumour cells, thereby enhancing the spatial and temporal accuracy of drug action. These characteristics of the localisation, release, and control of ethosomes render the system a highly viable nanocarrier for non-invasive therapy of skin cancer, achieving high efficacy with minimal toxicity. However, the targeting efficiency of ethosomes remains largely passive and less precise than that of fully designed nanocarriers, unless they are functionalised with ligands. Moreover, differences in receptor expression between tumour types and patients could influence the consistency of targeting. These aspects highlight the importance of individualised and flexible delivery plans [40, 54] (Table 3).

**Table 3:** Precision targeting mechanisms of ethosomes in skin cancer therapy

Targeting mechanism	Ethosome feature/modification	Mechanism of action	Therapeutic advantage	Reference
Passive targeting	Phospholipid bi-layer is flexible and contains high ethanol content	Ethanol liquidises stratum corneum lipids; the vesicles rupture to enter smaller intercellular spaces and put drugs into epidermis/dermis	High concentrations of the drug in the immediate environment of the tumour site, an elevated rate of drug excretion and retention, and limited systemic absorption	[50, 55]
Active targeting (ligand functionalization)	Surface ligands (e.g., RGD peptides, antibodies, folate, hyaluronic acid)	Ligand binds receptors on tumour cells, which were overexpressed on cell surfaces → receptor-mediated endocytosis → increased intracellular uptake	Specificity in the delivery to cancer cells, an increase in cytotoxicity, and reduced effects on normal cells	[52, 56]
Cancer versus normal cell selectivity	Takes advantage of differences in the TME of pH, receptor expression, and membrane fluidity	Selective internalisation by malignant cells because of a change in their membrane dynamics or relationship with ligands	Greater therapeutic index, decreases collateral toxicity of normal skin	[50, 57]
Sustained & controlled drug release	Encapsulation of lipid vesicles + stabilised with ethanol	Slowly released into the cell after having been endocytosed; shielded against extracellular degradation	Extensive exposure of tumour cells to drugs reduces off-target effects and systemic toxicity	[50]
TME targeting	Combination of immunomodulators and chemotherapeutics	Invasion of hypoxic/immune suppressive tumour areas regulates local communication	Increases tumour responsiveness, facilitates TME reprogramming, and decreases recurrence	[58]

## Ethosome-mediated reprogramming of tumour microenvironment

### Modulation of inflammatory cytokines

A persistent inflammation is one of the characteristics of skin cancer TME, which is elicited by high concentrations of pro-inflammatory cytokines (TNF-α, IL-1β, IL-6 and TGF- β). These cytokines stimulate tumour cell growth, epithelial-mesenchymal transition (EMT), immune evasion and angiogenesis. Anti-inflammatory or anticancer drugs can be delivered using ethosomes to achieve high drug concentrations in tumour-bearing skin, and cytokine-mediated signalling pathways can be suppressed [59]. Ethosomes can reduce chronic inflammation and break the feedback loop between cancer cells and inflammatory stromal cells by increasing drug residence time in the deeper epidermal and dermal layers. It has been established that nanocarrier technology can significantly decrease cytokine expression in the TME, effectively converting the local environment from a tumour-promoting into a tumour-suppressive state. This localised cytokine inhibition is especially useful in skin cancers; one does not want to immunosuppress a whole body [6].

### Anti-angiogenic effects

Angiogenesis of tumours, the development of new blood vessels, is another characteristic of cancer development that provides growing tumours with oxygen and nutrients and allows them to metastasise. Angiogenic inhibitors such as VEGF and FGF are useful for starving tumours and normalising irregular vasculature. Anti-angiogenic drugs, photosensitizers, or small-molecule disruptions of angiogenic signalling can be formulated into ethosomes. Ethosomal photodynamic therapy (PDT) preparations have been shown to reduce tumour angiogenesis and proliferation in squamous cell carcinoma (SCC) models by delivering photosensitizers into deep tumour tissue and inducing vascular damage and cell death upon light activation. Even though this is a localised phenomenon, targeted delivery can diminish neovascular supply to the tumour and reconstitute the microenvironmental angiogenic environment. Also, nanomedicine strategies in general, such as ethosomal systems, can be tailored to deliver anti-angiogenic factors that locally suppress pro-angiogenic factors in the areas of interest, thereby inhibiting the formation of new vessels and tumour progression [60, 61].

### Immune microenvironment remodelling

Immunosuppressive cells present in the TME include regulatory T cells (Tregs) and tumour-associated macrophages (TAMs), which suppress effective antitumour immunity. The ability to remodel this immune microenvironment to support effector T cells and pro-inflammatory macrophages (M1 phenotype) is important for responding to therapy. Nanocarrier delivery platforms can co-deliver immune checkpoint inhibitors or immunomodulators into the TME, induce local immune activation, and minimise global side effects. Although engineered exosomes or lipid nanoparticles are used in the majority of studies on immune remodelling, the enhanced skin penetration and local drug delivery afforded by ethosomes offer an opportunity to deliver immunomodulatory agents (e.g., TLR agonists, checkpoint modulators) into the cutaneous TME to enhance local antigen presentation and immune activation. This local delivery is useful in skewing the immunosuppressive TME to an immuno-responsive one [62, 63].

### Delivery of siRNA, miRNA, and gene-based therapeutics

Small interfering RNA (siRNA) and microRNA (miRNA) are gene-based therapeutic options with potential for reprogramming the TME and silencing oncogenic pathways. The instability and rapid degradation of these nucleic acids, together with poor cellular uptake, make it difficult to deliver the nucleic acids into the tumour. RNA therapy can be stabilised by nanotechnology and delivered to the target cells more effectively. Even though the majority of siRNA/miRNA delivery literature centres on lipid nanoparticles or extracellular vesicles (exosomes), ethosomal systems are also highly applicable to this concept: ethosomes can encapsulate nucleic acids or gene-editing constructs to produce localised gene silencing or modulation of TME pathways in skin tumours. Ethosomes may be modified to target siRNA/miRNA by internalising them into cells, to silence pro-tumourigenic genes by blocking extracellular degradation of nucleic acids, and to target immune checkpoints in the TME [64, 65]. In particular, miRNA-based antitumour responses and evasion of resistance pathways via nanocarrier-mediated delivery of miRNAs targeting tumour-associated macrophage polarisation or immune-suppressive cytokines have been demonstrated, and similarly, approaches based on ethosomal platforms can be developed [66].

## Therapeutic payloads for TME-targeted skin cancer therapy

### Cytotoxic and targeted anticancer agents

The main mechanism of action of cytotoxic and molecularly targeted anticancer agents is DNA damage, cell cycle arrest, and induction of apoptosis in cancer cells. There is, however, an emerging body of evidence suggesting that these agents also affect the TME by modulating cytokine release, stromal cell activity, and growth factor signalling. In non-invasive skin cancer, aberrant activation of pathways including Hedgehog (basal cell carcinoma), MAPK, PI3K/Akt, and EGFR is important for tumour maintenance. Precise anticancer drugs inhibit these pathways, resulting in tumour cell death and reducing the secretion of pro-tumour mediators such as VEGF, TGF-β, and IL-6 in the TME. Moreover, the localised anticancer therapy disturbs the tumour cell-fibroblast crosstalk and interferes with the extracellular matrix remodelling and mechanical support of tumour growth. The biological benefit of localised, skin-confined therapy is that it ensures prolonged antitumour pressure and reduces systemic immunosuppression and toxicity, especially in elderly and immunocompromised individuals [67, 68].

### Natural bioactives and tumour-suppressive phytochemicals

The unique features of natural bioactive compounds as anticancer agents are their pleiotropic biological action and the capacity to simultaneously control multiple cancer hallmarks. However, in contrast to traditional cytotoxic drugs that act directly on tumour cells, phytochemicals have a significant impact on the TME by inhibiting chronic inflammation, oxidative stress, angiogenesis, and immune dysfunction. These compounds suppress key transcription factors, including NF-κB, STAT3 and AP-1, which are major promoters of inflammatory cytokine production and tumour-stimulating immune responses. Persistent UV-induced inflammation in skin cancer forms a pro-tumour niche rich in reactive oxygen species and immunosuppressive cytokines. Phytochemicals respond to this environment by maintaining redox homeostasis, blocking matrix metalloproteinase and inducing tumour cell differentiation. Notably, they have immunomodulatory activities that increase antigen presentation and promote immune surveillance in the cutaneous microenvironment, and are therefore highly applicable to long-term tumour control and chemoprevention regimens [69, 70].

### Photodynamic therapy-associated therapeutic agents

Photodynamic therapy (PDT) is a developed non-invasive modality that is specifically appropriate for superficial and early-stage skin cancer. Its treatment effect goes way beyond localised destruction of tumour cells. Upon light activation, PDT generates reactive oxygen species that destroy tumour vasculature, disrupt oxygen homeostasis, and induce immunogenic cell death. The events result in the release of tumour-associated antigens and damage-associated molecular patterns (DAMPs), thereby enhancing the recruitment and activation of immune cells in the TME [71]. This also applies to PDT, which exhibits anti-angiogenic properties by preferentially destroying endothelial cells in tumour-related blood vessels, thereby causing vascular collapse and nutrient deprivation. This restructuring of the TME and immune-mediated clearance of residual tumour cells is achieved by this vascular modulation. Thus, PDT-associated agents serve as microenvironment-scale modulators that mediate tumour ablation and immune activation during non-invasive treatment of skin cancer [72, 73].

### Nucleic acid-based and gene regulatory therapeutics

Gene dysregulation initiates both tumour initiation and microenvironmental remodelling in skin cancer. Nonic acid Therapeutic uses offer unparalleled selectivity in controlling oncogenes, tumour suppressors, immune checkpoints, and angiogenic mediators. These agents directly intervene to control the behaviour of tumour cells by silencing oncogenic drivers or reinstating tumour-suppressive genes, and concomitantly reprogram stromal and immune components of the TME. Regulation of gene therapeutics is also notable for immune reactivation, targeting immune checkpoint molecules and macrophage polarisation pathways. Localised gene modulation during non-invasive skin cancer therapy eradicates systemic genetic risks and provides spatial regulation of therapeutic delivery. This precision is consistent with the novelties of personalised and molecularly-targeted dermatological oncology [74, 75].

### Synergistic and combination therapeutic strategies

Adaptive resistance and tumour heterogeneity are some of the biggest challenges to the successful control of skin cancer. Combination therapeutic strategies seek to address these issues by acting upon tumour cells and simultaneously several TME components. Combination treatment technologies inhibit compensatory survival mechanisms and enhance therapeutic durability by combining cytotoxic agents with immunomodulatory, gene-regulatory, or microenvironment-normalising therapies. Biologically, combination therapy allows simultaneous interference with tumour proliferation, angiogenesis, immunosuppression and stromal support. In non-invasive skin cancer, localised combination therapy is used to lower drug doses, enhance safety, and improve patient compliance. These methods reflect the principles of precision medicine by addressing tumour complexity and not relying on single-target interventions [76, 77].

## Clinical relevance in non-invasive skin cancer management

### Potential role in basal cell carcinoma and squamous cell carcinoma

The most common types of non-melanoma skin cancer include basal cell carcinoma (BCC) and squamous cell carcinoma (SCC), which are normally localised to the epidermal and superficial dermal layers in their initial stages, which are non-invasive. This locality renders them very receptive to transdermal drug delivery methods. An ethosome-based drug delivery system is also a promising therapeutic alternative, as it provides deep skin penetration and prolonged localisation of the drug at the tumour site without exposure to the body’s systems. Preclinical testing showed that ethosome-encapsulated anticancer agents are more likely to achieve higher intratumoural drug concentrations in a BCC and SCC model than traditional topical preparations, leading to increased cytotoxicity against malignant keratinocytes. Also, ethosomes may be used to achieve specific changes in the tumour microenvironment, such as inhibiting inflammatory mediators and angiogenic signalling pathways that play key roles in tumour progression in BCC and SCC. This makes ethosomal therapy a possible non-invasive or supplemental modality of therapy [78, 79].

### Clinical advantage over surgical and ablative approaches

Standard care for most non-invasive skin cancers includes surgical excision, Mohs micrographic surgery, and ablative therapies, although these are not associated with scarring, infection risk, or functional impairment. Cosmetics are considered sensitive, such as the face or neck. Topical therapy based on ethosomes is a non-invasive treatment method with minimal tissue injury and therapeutic effect. Compared with ablative methods, ethosomal formulations provide controlled, localised drug delivery, which minimises collateral injury to surrounding healthy skin. Additionally, repeated dosing can be performed using ethosomes, which would not cause tissue trauma over time, which is especially beneficial in cases of multifocal lesions or recurring BCC and SCC. The fact that they can reprogramme the TME also contributes to improved therapeutic outcomes, as it eliminates tumour regrowth and resistance, which are common with purely destructive therapies [80, 81].

### Patient compliance, cosmetic outcomes, and quality of life

Quality of life and patient adherence are important factors in the treatment of chronic and recurrent skin cancer. Topical ethosome-based formulations are usually painless, easy to apply, and non-irritating, with better patient compliance than invasive procedures. Significantly, ethosomal therapies maintain skin architecture, leading to better cosmetic outcomes with minimal scarring or pigmentary alterations. This is especially so in the case of elderly patients and those with multiple lesions that need long-term care. Patient-reported outcomes have been assessed in research, and it was noted that a non-invasive topical nanocarrier system significantly enhances a sense of contentment, psychological well-being, and general quality of life. Ethosome-mediated therapy is consistent with patient-centred models of cancer care by reducing treatment-related pain and improving aesthetics [82, 83].

### Challenges in clinical adoption

Although there is evidence of encouraging preclinical results, ethosome-based systems have not yet been translated into clinical use due to several critical issues. These include variability in the permeability of human skin, the absence of standardised formulation guidelines, inadequate large-scale clinical evidence, and concerns about the long-term safety of repeated ethanol exposures. Moreover, regulatory uncertainty surrounding nanocarrier-based topical systems further delays approval processes. The way forward for ethosomes to be translated from experimental platforms into therapies with clinical approval rests on addressing these translational gaps [84].

## Limitations and challenges

Although ethosomal systems have the potential to be major advancements in the delivery of therapeutic drugs through the skin, there are several important drawbacks that prevent their clinical application. A significant issue is that ethosomes are physicochemically unstable, mainly because of the high concentration of ethanol, which may cause the vesicles to rupture, allow intravesicular drug to leak out, and shorten shelf life during storage. Moreover, formulations containing higher ethanol concentrations can lead to skin irritation, erythema, and barrier disruption, which is also a concern for safety and patient compliance, especially when used over the long term [89, 90]. Moreover, mass production is difficult because formulation parameters, including ethanol concentration, lipid composition, and processing conditions, are sensitive, leading to a lack of reproducibility and batch-to-batch variability. Despite positive in vitro and preclinical studies, there is a significant gap due to the lack of clearly established clinical evidence demonstrating its safety and effectiveness in humans. Furthermore, economic factors, as well as the lack of clear regulatory measures of the nanocarrier-based systems, also restrain their commercialisation. Thus, although ethosomal systems have high potential for experimentation, they require a high level of optimisation in terms of stability, scalability, safety, and regulatory standardisation before successful clinical use [10–88].

## Safety, toxicological, and ethical considerations

### Local skin tolerance and long-term safety concerns

Topical drug delivery systems involving ethosomes are widely regarded as biocompatible because of their phospholipid composition, but the high ethanol content raises significant concerns about local skin tolerance. Temporal experiments have indicated that ethosomal formulations are tolerable for topical use, with a low incidence of erythema or irritation compared with traditional penetration enhancers. However, with chronic and frequent use, especially in chronic disease treatment like non-invasive skin cancers, skin barrier homeostasis, lipid organisation, and skin hydration can change. Long-term exposure to ethanol-containing vesicles may lead to subclinical inflammation or impairment of epidermal integrity, particularly in older or fragile patients. Thus, long-term dermatotoxicity research is all that is required to determine the safety of ethosome-mediated treatments for repeated utilisation in clinical practice [85].

### Nanocarriers associated with biological risks

Beyond effects on the local skin, nanocarrier-based systems pose distinctive biological risks due to their size, surface characteristics, and interactions with biological membranes. The nanoscale size of ethosomes has been shown to enable deeper penetration into the skin and exposure to immune cells, fibroblasts, and endothelial cells. Although this property is beneficial for tumour targeting, non-target cellular uptake or accumulation can lead to oxidative stress, inflammatory responses, or disrupted cellular signal transduction. Also, there is a limitation in the description of the future of nanovesicles after cellular internalisation and long-term biodegradation patterns. It is especially relevant to address these nanotoxicological issues by using ethosomes to deliver potent anticancer or gene-based therapeutics in the treatment of skin cancer [86].

### Ethical considerations in nanomedicine-based skin therapy

The application of nanotechnology in dermatological oncology raises several ethical issues, particularly regarding patient safety, informed consent, and risk-benefit analysis. Since ethosome-based therapies are a new treatment modality, patients should have sufficient information on the possible unknown risks of long-term exposure. There are also ethical issues regarding fair access to novel nanomedicine-based therapies, which may be expensive or available only in specialised facilities. Moreover, gene-based therapies as well as immune-modulating therapies based on nanocarriers require close ethical controls to avoid abuse or unexpected biological effects. Clinical reporting and ethical review systems should then be transparent to conduct responsible clinical translation [85].

### Regulatory perspectives for topical nanotherapeutics

Topical nanotherapeutics like ethosomes still face a complex and evolving regulatory assessment landscape. Traditional regulatory frameworks are not always sufficient to address the physicochemical and biological peculiarities of nanocarriers. The main regulatory issues are the standardisation of characterisation parameters, the evaluation of dermal absorption, long-term toxicity, and batch-to-batch reproducibility. The US FDA and EMA have highlighted the need for case-by-case risk evaluation of nanomedicine-based products. In the case of ethosome-based therapies of skin cancer, there must be specific regulatory guidelines to enable such therapies to be approved clinically whilst remaining safe to the patients. The standardisation of international regulatory standards will be a critical factor in accelerating the adoption of clinical standards [87].

## Emerging trends and future directions

### Integration with immunotherapy and molecular targeting

Recent developments in the treatment of skin cancer have put the central role of immune modulation and molecularly targeted therapies at the forefront of managing tumour growth and preventing recurrence. The delivery system of ethosomes is a promising approach for incorporating topical immunotherapeutic agents and molecular inhibitors into the tumour microenvironment. With the ability to enable efficient dermal penetration and localised delivery, ethosomes can be utilised in providing topical delivery of immune checkpoint modulators, cytokine regulators, and pathway-specific inhibitors of aberrant signalling cascades in basal and squamous cell carcinomas. Localised immunomodulation with such immunomodulators reduces systemic adverse immune effects and induces antitumour immunosurveillance in the skin. The combination of ethosomal delivery and immunotherapy is a plan to obtain long-term tumour control in non-invasive skin cancer [88, 89].

### Precision medicine and personalised skin cancer therapy

The goal of precision medicine is to personalise therapeutic interventions based on the unique tumour biology, genetic changes and microenvironmental features. Ethosomes provide a flexible delivery vehicle that can be modified to individualise the treatment of skin cancer by loading it with a patient-specific therapeutic payload. Lipid content, immunology, and inflammatory effects on tumour progression in non-invasive skin tumours can be addressed with tailor-made ethosomal preparations of specific target drugs or nucleic acid-based therapeutic agents. Moreover, site-specific, controlled delivery is possible with ethosomes, which can be very useful in the context of personalised oncology to achieve maximum efficacy and minimise off-target toxicity. With the evolving biomarker-based diagnosis, ethosome-mediated therapy is bound to be relevant in making precision dermatological oncology approaches [5, 90].

### Smart delivery concepts and adaptive therapeutics systems

The future of ethosome-based systems is shifting from passive delivery to smart and adaptive therapeutic systems. Ethosomes, triggered by stimuli to release their cargo of therapeutic molecules in response to tumour-specific signals such as changes in pH, oxidative stress, or inflammatory response enzymes, are becoming potent agents for selective therapy of skin cancer. These adaptive systems can dynamically alter drug release profiles in response to tumour microenvironmental conditions, improving therapeutic precision and minimising unnecessary exposure to healthy tissue. Smart ethosomal carriers can also be used for sequential/combination therapy delivery, allowing simultaneous tumour destruction and microenvironmental reprogramming in non-invasive skin cancers [91, 92].

### Artificial intelligence and data-driven optimisation

Artificial intelligence (AI) has a disruptive role during the rational design and optimisation of ethosomal drug delivery systems. AI integration design philosophy is informed by data-driven modelling. Data parameters such as vesicle size, zeta potential, lipid composition, ethanol concentration, and drug characteristics are the input variables used to predict formulation performance. The algorithms used in machine learning, including artificial neural networks (ANNs), support vector machines (SVMs), and random forests, are used to identify optimal formulation conditions and optimise drug loading, stability, and skin permeation efficiency. Regarding the principles of operation, AI models are trained on experimental data to determine correlations between formulation variables and treatment effects. These models are capable of predicting the drug release profile, penetration depth in the skin, and interactions with components of the tumour microenvironment. Moreover, image analysis using AI offers a valuable opportunity to detect and classify skin lesions and to provide personalised, targeted drug administration. The combination of AI and ethosomal systems facilitates precision medicine by enabling the development of adaptive formulation strategies personalised to patient profiles. Although it has its benefits, issues such as data availability, model validation, and clinical translation are of great concern for successful implementation. Design-wise, AI-based ethosomal systems are grounded in a data-centric modelling approach, in which formulation parameters, biological interactions, and therapeutic effects are interrelated through predictive algorithms. These systems are based on iterative learning frameworks that continuously optimise formulation efficiency using experimental and clinical data, thereby enabling adaptive and intelligent drug delivery design [93, 94].

## Conclusion

Ethosomal drug delivery systems are a recent development in transdermal therapeutics, with the potential to enhance drug permeation and bioavailability. Ethanol and flexible phospholipid vesicles enable effective penetration through the stratum corneum, circumventing the limitations of traditional topical formulations. Nevertheless, several challenges remain to be overcome before they can be successfully translated into clinical use. Vesicle instability, potential skin irritation due to high ethanol concentrations, the lack of large-scale production methods, and limited clinical information are among the issues that limit their use. Moreover, ethosomes exhibit better permeation than other nanocarrier systems such as liposomes and niosomes, yet may be limited in stability and cost-effectiveness. Thus, future research must focus on enhancing formulation stability, reducing side effects, and conducting large-scale clinical trials to determine safety and effectiveness. Also, scalable manufacturing and regulatory standardisation should be developed to ensure commercialisation. In general, although ethosomes have great potential, a more rigorous and methodical approach is needed to bring this technology out of the laboratory and into clinical practice.

## Data Availability

No datasets were generated or analysed during the current study.
